# Predicting HIV-1 Protease Cleavage Sites With Positive-Unlabeled Learning

**DOI:** 10.3389/fgene.2021.658078

**Published:** 2021-03-26

**Authors:** Zhenfeng Li, Lun Hu, Zehai Tang, Cheng Zhao

**Affiliations:** ^1^School of Computer Science and Technology, Wuhan University of Technology, Wuhan, China; ^2^Xinjiang Technical Institute of Physics and Chemistry, Chinese Academy of Sciences, Ürümqi, China

**Keywords:** HIV-1 protease, cleavage site prediction, positive-unlabeled learning, biased SVM, substrate specificity

## Abstract

Understanding the substrate specificity of HIV-1 protease plays an essential role in the prevention of HIV infection. A variety of computational models have thus been developed to predict substrate sites that are cleaved by HIV-1 protease, but most of them normally follow a supervised learning scheme to build classifiers by considering experimentally verified cleavable sites as positive samples and unknown sites as negative samples. However, certain noisy can be contained in the negative set, as false negative samples are possibly existed. Hence, the performance of the classifiers is not as accurate as they could be due to the biased prediction results. In this work, unknown substrate sites are regarded as unlabeled samples instead of negative ones. We propose a novel positive-unlabeled learning algorithm, namely PU-HIV, for an effective prediction of HIV-1 protease cleavage sites. Features used by PU-HIV are encoded from different perspectives of substrate sequences, including amino acid identities, coevolutionary patterns and chemical properties. By adjusting the weights of errors generated by positive and unlabeled samples, a biased support vector machine classifier can be built to complete the prediction task. In comparison with state-of-the-art prediction models, benchmarking experiments using cross-validation and independent tests demonstrated the superior performance of PU-HIV in terms of AUC, PR-AUC, and F-measure. Thus, with PU-HIV, it is possible to identify previously unknown, but physiologically existed substrate sites that are able to be cleaved by HIV-1 protease, thus providing valuable insights into designing novel HIV-1 protease inhibitors for HIV treatment.

## 1. Introduction

As the causative agent of acquired immunodeficiency syndrome (AIDS), human immunodeficiency virus type 1 (HIV-1) is able to destroy the immune system of human body by spreading in a cell-free system or from cell to cell (Abela et al., 2012). A series of laboratory-based experiments have been conducted in order to better understand the mechanisms of HIV-1 replicative cycle. Their results indicate that HIV-1 protease (PR) plays an essential role in producing mature and infectious virions (Sadiq et al., 2012). In particular, HIV-1 PR guarantees the maturation of HIV virions by cleaving the viral precursor Gag and Gag-Pol polyproteins into infectious virus particles with aberrant structure (Weber et al., 1989). Hence, for the purpose of HIV treatment, an efficient way is to prevent the HIV-1 replication by inhibiting the activity of corresponding PR.

A variety of HIV-1 PR inhibitors have thus been developed. Due to their capability of tightly binding to HIV-1 PR, these PR inhibitors make it possible for peptide substrates to avoid being cleaved by HIV-1 PR. Obviously, the substrate specificity of HIV-1 PR is of great significance for designing novel and reliable HIV-1 PR inhibitors, thus considerably minimizing the side effects caused by them (Devroe et al., 2005). Certain efforts have been made by laboratory-based experiments to identify the cleavage sites targeted by HIV-1 PR. Although promising, they have the disadvantages of being time-consuming and labor-intensive (Wagner et al., 2015). Moreover, since both sequence homology and binding motif are rarely observed for the cleavage sites in the viral polyproteins (Kontijevskis et al., 2007), the substrate specificity of HIV-1 PR could be only partially understood at present. In this regard, the problem of effectively identifying HIV-1 PR cleavage sites is still challenging.

With the development of machine learning techniques in the last few decade years, many computational models have been proposed to overcome the aforementioned disadvantages of laboratory-based experiments from an alternative view. These models normally consider the identification of HIV-1 PR cleavage sites as a typical prediction problem, which is then addressed by integrating different features of substrate sequences with various classifiers. Although the substrate specificity of HIV-1 PR is more likely to be determined by shape complementarity between substrates and HIV-1 PR instead of simply relying on a particular amino acid sequence (Prabu-Jeyabalan et al., 2002), the sequence information of substrates are primarily adopted to perform the prediction task due to its wide availability, low cost and generally satisfactory performance especially for the large-scale prediction of HIV-1 PR cleavage sites.

Since HIV-1 protease specifically binds with a precursor protein in octapeptide length before cleavage, susceptible sites in a substrate are considered as octapeptide regions, each of which is sequentially composed by eight amino acids. Two problems have to be addressed for achieving an accurate prediction of HIV PR cleavage sites. The first problem is feature extraction where a set of relevant features is generated to encode octamer sequences, and the other one is the selection of an appropriate classifier used to determine the substrate specificity of HIV-1 PR. Existing prediction models develop different solutions to address these two problems, and some representative works are presented as follows.

In the earlier stage, many researchers concentrated on employing different classification models to predict HIV-1 PR cleavage sites. In particular, Thompson et al. (1995) applied an artificial neural network (ANN) with a standard feed-forward multilayer perceptron to classify cleavage sites from a small set of octapeptides. Narayanan et al. (2002) constructed a decision tree model to extract useful rules for HIV-1 PR cleavage site prediction, but found that the performance of decision tree was not as well as that of ANN. After that, Cai et al. (2002) made use of support vector machine (SVM) with different kernels to solve the prediction problem, and the experiment results indicated that among all kernels a Gaussian kernel yielded the best accuracy. Kontijevskis et al. (2007) integrated rough set theory with genetic algorithms to extract rules related to the existence of cleavage sites and concluded that a cleavage event occurred with a greater chance if at least three amino acids were combined in the substrate. As a web-server, HIVcleave (Shen and Chou, 2008) was established by combining discriminant function algorithm and vectorized sequence-coupling model. One should note that most of previous works considered the prediction of HIV-1 PR cleavage sites as a non-linear problem, but Rögnvaldsson and You (2004) argued that this problem ought to be linear and could be solved with a simple linear model, such as linear SVM (LSVM). A possible reason for the lack of evidence supporting the non-linear nature of the prediction problem was ascribed to the insufficient data used for training and testing.

Regarding the linear separability observed in the octamer sequences, the problem of how to encode octapeptides with features distinguishing such separability has attracted much attention recently. Gök and Özcerit (2013) used the OETMAP coding scheme based on amino acid features and integrated it with a linear classifier. An cross-validation comparison with standard coding schemes was performed and the experiment results showed that the OETMAP coding scheme improved the prediction performance. Rögnvaldsson et al. (2015) further adopted a LSVM model combined with orthogonal coding, and claimed that the proposed model achieved a better performance in predicting the cleavage sites of HIV-1 protease when compared with state-of-the-art prediction models. PROSPERous (Song et al., 2018) was developed as a reliable integrated system by using different scoring functions to construct feature vectors for octapeptides. iProt-Sub (Song et al., 2019) integrated heterogeneous sequence and structural features, and then used a two-step feature selection procedure to further remove redundant and irrelevant features, thereby improving the prediction accuracy of cleavage sites. As the latest attempt in this direction, Hu et al. (2020a) proposed an novel feature extraction method, namely EvoCleave, to identify coevolutionary patterns from substrate sequences with the ability of providing certain evidence to support or refute the existence of cleavage site in a substrate. DeepCleave (Li et al., 2020) used deep learning to extract high-quality cleavage site features from protein substrate sequences, and employed a convolutional neural network with transfer learning function to train prediction models.

Most of prediction models mentioned above usually construct a binary classification model by considering cleavable and non-cleavable octapeptides as positive and negative training sets, respectively. Although cleavable octapeptides are experimentally confirmed, non-cleavable octapeptides are artificially generated by adopting different strategies. For example, Rögnvaldsson et al. (2015) shifted two positions toward either side of the cleavage site along the peptide and labeled the resulting octamers as non-cleavable. Obviously, the artificial generation of non-cleavable octapeptides are prone to encounter false negative results, which in return degrade the prediction accuracy of classifiers. In this regard, the classification models built on the positive and noisy negative training sets are not capable of being accurate as they could in predicting previously unknown, but physiologically existed HIV-1 PR cleavage sites.

Rather than composing a negative training set with unknown octapeptides, we treat them as an unlabeled set for training. In this work, a novel positive-unlabeled (PU) prediction model, namely PU-HIV, is proposed to build a more accurate classifier for predicting HIV-1 PR cleavage sites based on positive and unlabeled training sets. To do so, PU-HIV first uses a comprehensive combination of amino acid identities, coevolutionary patterns and chemical properties to represent octapeptides into feature vectors. Such a combination allows PU-HIV to fully exploit the sequence information of substrates for the prediction task. Then, PU-HIV adopts a biased formulation of LSVM to distinguish the penalties of incorrectly classifying positive and unlabeled octapeptides, as an accurate classifier can be constructed by maximizing the number of unlabeled examples classified as negative while constraining the positive examples to be correctly classified according the PU learning theory (Liu et al., 2002). Finally, a Biased SVM classifier is built by using positive and unlabeled training sets for predicting novel HIV-1 PR cleavage sites.

As the first attempt in applying PU learning for HIV-1 PR cleavage site prediction, PU-HIV has been compared with several state-of-the-art prediction models, including HIVcleave (Shen and Chou, 2008), Rögnvaldsson et al. (2015), PROSPERous (Song et al., 2018), iProt-Sub (Song et al., 2019), EvoCleave (Hu et al., 2020a), and DeepCleave (Li et al., 2020). Experiment results demonstrated the promising accuracy of PU-HIV, as it yielded the best scores of AUC, PR-AUC, and F-measure across almost all datasets used for evaluation. Hence, we reason that the novel HIV-1 PR substrate sites predicted by PU-HIV are able to provide valuable insights into designing new HIV-1 PR inhibitors for HIV treatment.

## 2. Materials and Methods

**Figure 1:**
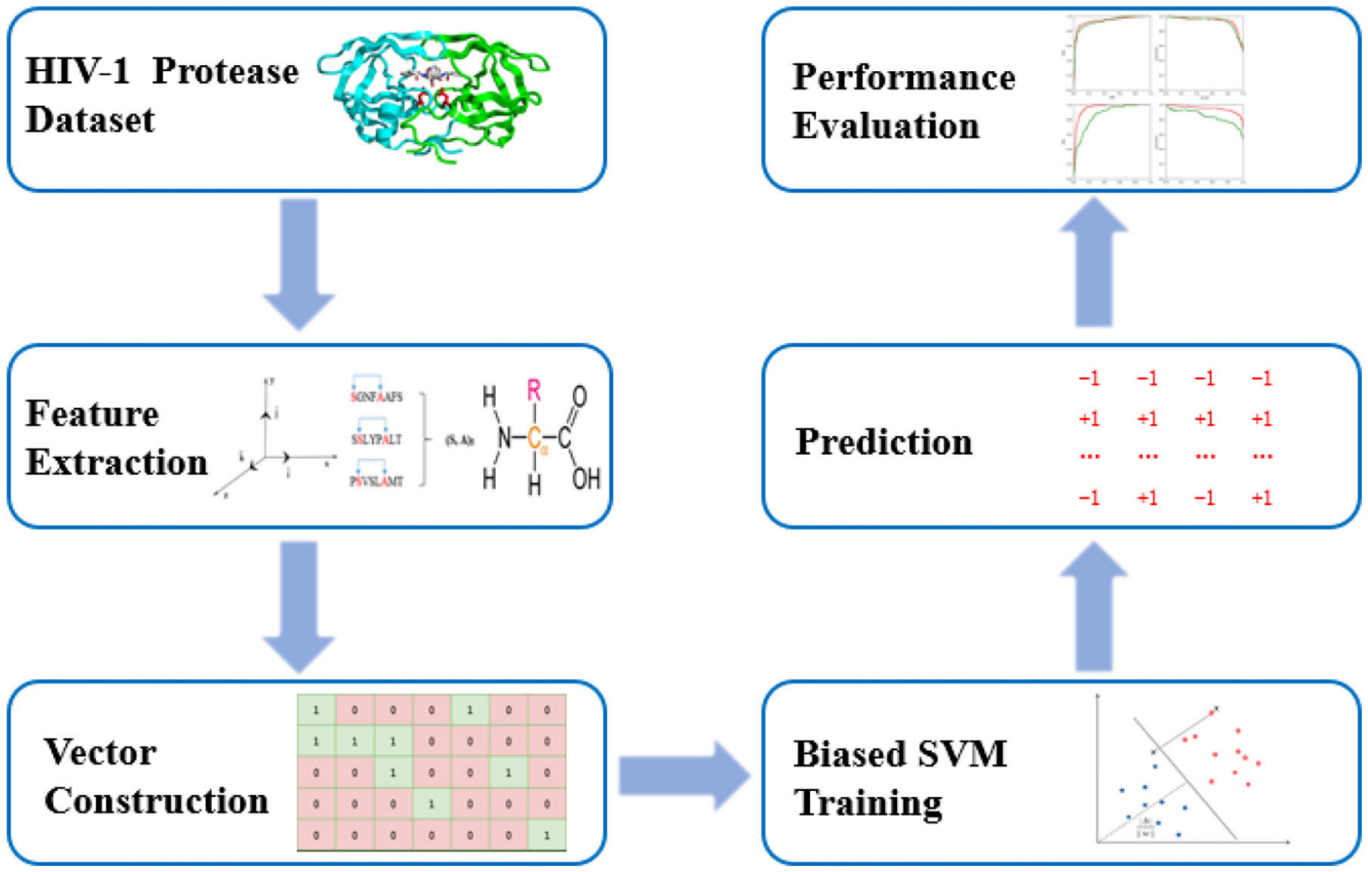
The workflow of PU-HIV.

PU-HIV is composed of three steps including feature vector construction, biased SVM classifier training and prediction. In particular, PU-HIV makes use of a combination of amino acid identities, coevolutionary patterns, and chemical properties to construct the feature vectors of octapeptides. After that, a biased SVM classifier is built to predict novel HIV-1 PR substrate sites based on positive and unlabeled training sets, and lastly its performance is evaluated with several metrics. The workflow of PU-HIV is presented in Figure 1.

### 2.1. Experimental Datasets

To avoid any potential bias resulted from the selection of training and testing sets in performance evaluation, we selected five benchmarking datasets denoted as 301Dataset, 746Dataset, 1625Dataset, impensDataset, and schillingDataset, respectively. All these five datasets are available to be downloaded from the UCI machine learning repository (Dua and Graff, 2019). A detailed description about each of datasets is presented in Table 1. We noted that all the datasets except for 746Dataset were imbalanced, as the size of positive set was much less than that of unlabeled set. Obviously, such an imbalanced distribution is able to significantly manipulate the training process of a classifier, thus affecting the predictive accuracy. Moreover, the robustness of PU-HIV could also be indicated by its performance on these datasets.

**Table 1:** Detailed descriptions of five datasets.

**Dataset**	**References**	**Total**	**P**	**U**
301Dataset	Chou (1996)	301	62	239
746Dataset	You et al. (2005)	746	401	345
1625Dataset	Kontijevskis et al. (2007)	1,625	374	1,251
impensDataset	Rögnvaldsson et al. (2015)	947	149	798
schillingDataset	Rögnvaldsson et al. (2015)	3,272	434	2,838

*The column of References gives the original source of corresponding dataset. The column of Total is the number of all octapeptides in the dataset. The columns of P and U are the sizes of positive and unlabeled sets, respectively*.

### 2.2. Feature Vector Construction

Each octapeptide is a sequence composed of eight amino acids. In particular, given an alphabet set Λ = {λ_*i*_}(1 ≤ *i* ≤ *n*_Λ_, *n*_Λ_ = 20) representing a set of 20 distinct amino acids, an octapeptide is represented as $P=P_{4}P_{3}P_{2}P_{1}P_{1}^{\prime }P_{2}^{\prime }P_{3}^{\prime }P_{4}^{\prime }$ where $P_{i},P_{j}^{\prime }\in \Lambda (1\leq i,\;j\leq 4)$. The cleavage site is located between $P_{1}^{\prime }$ and *P*_1_. To fully exploit the sequence information of octapeptides, we decide to use a combination of three different features, i.e., amino acid identities, coevolutionary patterns and chemical properties, for feature vector construction. The details of extracting these features are presented as follows.

#### 2.2.1. Amino Acid Identities

To distinguish amino acids in the feature space, we adopt an orthogonal encoding scheme where each amino acid is represented by a orthogonal vector with 20 bits. Taking λ_*i*_ ∈ Λ as an example, the *i*-th element in its corresponding vector is set to 1 while the other elements are set to −1. In dong so, each octapeptide is then mapped to 160-dimensional vector in a sparse orthogonal representation. Moreover, since the value of the last element in an orthogonal vector is constrained by the values of the other seven elements, the dimensionality could further reduce from 160 to 152 by simplifying removing the constraints for all the eight amino acids in an octapeptide.

However, due to the shift-variant property of cleaved octapeptides, it is possible that the vectors of two cleaved octapeptides are located very distantly in the orthogonal space. Taking two octapeptides *ADIYTEHA* and *YSAFLVAD* as an example, these two octapeptides are verified to be cleaved in the schilling dataset, but their amino acids at the same position are different. In this regard, the Hamming distance between them is the largest in the orthogonal space, and accordingly it is difficult for classifiers to group them into the same category. To minimize the effect of shift-variance, we also incorporate the other two kinds of features into constructing the feature vectors of octapeptides.

#### 2.2.2. Coevolutionary Patterns

In HIV envelope proteins, the change in amino acid at one residue sometimes may give rise to the change at another residue (Travers et al., 2007). Motivated by this observations, EvoCleave targets to discover the knowledge of coevolving between pairwise amino acids that are capable of providing certain evidence to support or refute the existence of cleavage site in substrates by HIV-1 PR. Assuming that (_λ_*i*_, λ_*j*_)*k*_ denotes that λ_*i*_ is followed by λ_*j*_ at *k* − 1 positions later, EvoCleave determines whether (_λ_*i*_, λ_*j*_)*k*_ is a coevolutionary pattern by (1).

(1) $$\begin{array}{l} diff\left({\left(\lambda_{i},\lambda_{j}\right)}_{k}\right)=\\ \frac{p({\left(\lambda_{i},\lambda_{j}\right)}_{k})-p({\left(\lambda_{i},*\right)}_{k})p({\left(*,\lambda_{j}\right)}_{k})}{\sqrt{\frac{p({\left(\lambda_{i},*\right)}_{k})p({\left(*,\lambda_{j}\right)}_{k})}{n_{1}}(1-p({\left(\lambda_{i},*\right)}_{k}))(1-p({\left(*,\lambda_{j}\right)}_{k}))}} \end{array}$$

In the above equation, *p*((_λ_*i*_, λ_*j*_)*k*_), *p*((_λ_*i*_, *)*k*_), and *p*((*,_λ_*j*_)*k*_) are the respective probabilities of observing (_λ_*i*_, λ_*j*_)*k*_, λ_*i*_, and λ_*j*_ in octapeptides, and *n*_1_ is the number of octapeptides. The purpose of (1) is to verify whether (_λ_*i*_, λ_*j*_)*k*_ is significantly frequently observed. Hence, (_λ_*i*_, λ_*j*_)*k*_ is considered as a coevolutionary pattern at a confidence level of 95% if *diff*((_λ_*i*_, λ_*j*_)*k*_) ≥ 1.96. For each coevolution pattern, EvoCleave further utilizes (2) to quantify the amount of evidence provided by this pattern from the perspective of mutual information.

(2) $$\begin{array}{l} weight\left({\left(\lambda_{i},\lambda_{j}\right)}_{k}\right)=\\ \log\frac{p({\left(\lambda_{i},\lambda_{j}\right)}_{k})}{p({\left(\lambda_{i},*\right)}_{k})p({\left(*,\lambda_{j}\right)}_{k})}-\log\frac{p({\left(\lambda_{i},*\right)}_{k})-p({\left(\lambda_{i},\lambda_{j}\right)}_{k})}{p({\left(\lambda_{i},*\right)}_{k})(1-p({\left(*,\lambda_{j}\right)}_{k})} \end{array}$$

Given (1) and (2), EvoCleave is able to extract all coevolutionary patterns from a set of octapeptides. Assuming that there are total *n*_2_ coevolution patterns, each octapeptide, i.e., **P**, is then mapped to a *n*_2_-dimensional feature vector. For each element in the feature vector, its value is set to the weight of corresponding coevolutionary pattern if this pattern and 0 otherwise. One should note that since the negative set is considered as unlabeled in the PU learning, we only apply EvoCleave to extract the coevolutionary patterns from cleaved octapeptides.

#### 2.2.3. Chemical Properties

Apart from amino acid identities and coevolutionary patterns, the chemical properties of amino acids are additionally considered for feature vector construction. In particular, all amino acids in Λ can be divided into eight different groups according to their chemical/structural properties (Dang et al., 2008). The grouping information is shown in Table 2. Similar to the procedure of handling amino acid identities, each amino acid is mapped into an orthogonal vector with only 8 bits. Taking λ_*i*_ ∈ Λ as an example, if λ_*i*_ belongs to the *k*−th chemical group, the *k*−th (1 ≤ *k* ≤ 8) element in its corresponding vector is set to 1 while the other elements are set to −1. Hence, each octapeptide can be encoded with a 64-dimensional vector. By removing the eight constraints, the dimensionality could be further reduced from 64 to 56.

**Table 2:** The chemical classes to which the 20 amino acids belong.

**Chemical group**	**Amino acids**
Sulfur-containing	C, M
Aliphatic 1	A, G, P
Aliphatic 2	I, L, V
Acidic	D, E
Basic	H, K, R
Aromatic	F, W, Y
Amide	N, Q
Small hydroxy	S, T

Although both chemical properties and amino acid identities encode octapeptides by following the orthogonal encoding scheme, the major difference lying between them is that orthogonal vectors encoded by chemical properties could, to some extent, moderate the effect of shift-variance. Still taking the two octapeptides *ADIYTEHA* and *YSAFLVAD* as an example, we note that the fourth amino acids, i.e., Y and F, are in the same chemical group of Aromatic. Hence, the Hamming distance between them in the orthogonal space of chemical properties is not as large as in the orthogonal space of amino acid identities.

In sum, after combining the features of amino acid identities, coevolutionary patterns and chemical properties, we finally are able to construct a (208 + *n*_2_)-dimensional feature vector for each octapeptide. Next the details of how to apply a biased SVM classifier for the prediction task are presented.

### 2.3. Biased SVM Classifier

A classical SVM classifier is to select a hyperplane that can best separate the positive and negative examples. Even though it has been argued that a LSVM classifier can achieve a superior predictive performance (Rögnvaldsson et al., 2015), we are not able to apply a LSVM classifier to explicitly solve the PU learning problem involved in this work, as the objective function formulated for a LSVM classifier fails to distinguish the training errors resulted from positive and unlabeled octapeptides. It is for this reason that we adopt a biased formulation of LSVM to complete the prediction task of HIV-1 protease cleavage sites.

Assuming that *D* ={(*x*_*i*_, *y*_*i*_)}(1 ≤ *i* ≤ *n*) is the training set, where *x*_*i*_ denotes the feature vector of **P**_*i*_ and *y*_*i*_ ∈ {−1, 1} is the label of **P**_*i*_, the first *m* − 1 octapeptides are verified to be cleaved by HIV-1 PR and they are positive examples labeled as *y*_*i*_ = 1(1 ≤ *i* ≤ *m* − 1), while the rest are unlabeled octapeptides whose labels are set to *y*_*i*_ = −1(*m* ≤ *i* ≤ *n*). Following Glasmachers and Igel (2010), we introduce two soft margin parameters, i.e., *C*_1_ and *C*_2_, to indicate the different tolerances on the training errors generated by positive and unlabeled octapeptides, respectively. These two parameters are also capable of learning from noisy unlabeled set that might contain cleaved octapeptides. The biased formulation of SVM with two L1-norm soft margins is defined by (3).

(3) $$\begin{array}{l} Minimize:\;\frac{1}{2}\omega^{T}\omega +C_{1}\sum_{i=1}^{m-1}\xi_{i}+C_{2}\sum_{i=m}^{n}\xi_{i}\\ s.t.\;y_{i}(\omega^{T}x_{i}+b)\geq 1-\xi_{i},\;\xi_{i}\geq 0,\;i=1,2,\ldots ,n \end{array}$$

In (3), ω is the normal vector of hyperplane separating positive and unlabeled octapeptides, ξ_*i*_ refers to the corresponding slack variable used to calculate the error cost for each octapeptide, and *b* denotes the offset of hyperplane from the origin along ω. Based on the biased formulation of SVM, a biased LSVM can be built by incorporating the linear kernel function defined by (4) into (3).

(4) $$kernel(x_{i},x_{j})=x_{i}^{T}\cdot x_{j}$$

The performance of a biased LSVM can be fine-tuned by adjusting the values of *C*_1_ and *C*_2_, and we intend to assign a larger value to *C*_1_ and a smaller one to *C*_2_. There are two reasons for such an intention. First, a larger value of *C*_1_ means a greater penalty to false positive predictions, thus ensuring that positive examples, i.e., cleavable octapeptides, are correctly classified as much as possible. The second reason is that a smaller value of *C*_2_ is able to maximize the number of unlabeled octapeptides as non-cleavable, but does not reject the possibility of containing cleavable octapeptides in the unlabeled set.

Regarding the implementation of a biased LSVM, we adopt the svm.SVC library provided by the sklearn package (Pedregosa et al., 2011). When determining the values of *C*_1_ and *C*_2_, we additionally introduce a parameter β to control the difference between *C*_1_ and *C*_2_ by using (5).

(5) $$C_{2}=\frac{C_{1}}{\beta }$$

During the fine-tuning phase, the values of *C*_1_ and β are varied from the sets {0.03123, 0.0625, 0.125, 0.25, 0.5, 1, 2, 4, 8, 16, 32} and {2, 5, 10, 20, 30, 50, 100, 200}, respectively. Since β is larger than 1, the value of *C*_2_ is always less than *C*_1_ according to (5), thus satisfying our intention regarding the setting of *C*_1_ and *C*_2_. After evaluating all possible combinations of *C*_1_ and *C*_2_, we use the combination with the best performance as the final setting to train the biased SVM classifier for predicting HIV-1 PR cleavage sites.

## 3. Results

In order to evaluate the performance of PU-HIV, we conducted a series of extensive experiments and also compared PU-HIV with several state-of-the-art prediction models including EvoCleave (Hu et al., 2020a), Rögnvaldsson et al. (2015), HIVcleave (Shen and Chou, 2008), PROSPERous (Song et al., 2018), iProt-Sub (Song et al., 2019), and DeepCleave (Li et al., 2020). Among them, EvoCleave, Rögnvaldsson et al. (2015), HIVcleave, PROSPERous, and DeepCleave (Li et al., 2020) are sequence-based models while iProt-Sub integrates different sources of biological information to complete the prediction task.

### 3.1. Evaluation Metrics

Three independent evaluation metrics, i.e., the area under the receiver operating characteristics curve (AUC), the area under the Precision-Recall receiver operating characteristics curve (PR ROC), and F-measure, were adopted to evaluate the accuracy of each algorithm, and their brief descriptions are given as follows:

The receiver operating characteristics (ROC) analysis considers the prediction accuracy as a trade-off between sensitivity and specificity, and the area under the ROC curve (AUC) is commonly used as a summary measure of the ROC curve. The AUC scores are within the range [0, 1]. The prediction performance of an algorithm is better if the corresponding AUC score is closer to 1 and vice versa. Similar to the ROC analysis, the Precision-Recall ROC (PR ROC) analysis concentrates on the trade-off between precision and recall by drawing a curve of precision vs. recall given different thresholds. The PR-AUC scores are computed by trapezoidal integral for the area under the precision-recall curve. The reason why we additionally adopted PR-AUC was that according to Table 1, most of datasets were imbalanced, as the number of cleaved substrates was much less than that of uncleaved ones. The PR ROC analysis has shown to be better than ROC for imbalanced datasets (Davis and Goadrich, 2006).

F-measure is the harmonic mean of precision and recall, and it has been widely adopted for performance valuation in different bioinformatics applications (Hu et al., 2020b). Its definition is given as follows.

(6) $$\begin{array}{l} \text{ Precision}=\frac{TP}{TP+FP}\\ \text{ Recall}=\frac{TP}{TP+FN}\\ \text{F}-\text{measure}=\frac{2\times Precison\times Recall}{Precision+Recall} \end{array}$$

In (6), *TP* is the number of correctly predicted octapeptides in the positive set, *FP* is the number of unlabeled octapeptides predicted to be cleavable, and *FN* is the number of cleavable octapeptides predicted to be uncleavable. In the experiments, the F-measure scores were computed at 50% threshold. In other words, an octapeptide is predicted to be cleavable if its probability obtained by PU-HIV is larger than 0.5.

### 3.2. 10-Fold Cross Validation

Results of the 10-fold cross validation (CV) experiment are presented in Table 3. In particular, each dataset was randomly divided into 10 equal-sized parts, we then alternatively used nine parts to train the PU-HIV classifier and evaluated it with the rest part.

**Table 3:** Experiment results of 10-fold CV.

**Dataset**	**Model**	**AUC**	**PR-AUC**	**F-measure**		
				**Precision**	**Recall**	**F-measure**
301Dataset	PU-HIV	0.96	**0.89**	0.87	0.76	**0.81**
	PU-HIV with standard SVM	0.96	0.88	0.86	0.77	**0.81**
	EvoCleave	0.91	0.81	0.37	0.94	0.53
	Rögnvaldsson et al. (2015)	0.93	0.86	0.85	0.74	0.79
	PROSPERous	0.94	0.45	0.21	1	0.34
	HIVcleave	**1**	0.61	1	0.55	0.71
	iProt-Sub	0.78	0.53	0.63	0.32	0.43
	DeepCleave	0.45	0.2	0.13	0.19	0.16
746Dataset	PU-HIV	**0.96**	**0.95**	0.91	0.91	**0.91**
	PU-HIV with standard SVM	0.94	0.93	0.89	0.87	0.88
	EvoCleave	0.93	0.92	0.9	0.8	0.85
	Rögnvaldsson et al. (2015)	0.92	0.91	0.85	0.9	0.87
	PROSPERous	0.84	0.53	0.54	1	0.7
	HIVcleave	0.74	0.81	0.92	0.7	0.8
	iProt-Sub	0.7	0.71	0.71	0.25	0.37
	DeepCleave	0.44	0.49	0.41	0.14	0.21
1625Dataset	PU-HIV	**0.98**	**0.95**	0.9	0.9	**0.9**
	PU-HIV with standard SVM	**0.98**	0.94	0.89	0.86	0.88
	EvoCleave	0.93	0.84	0.85	0.74	0.8
	Rögnvaldsson et al. (2015)	0.97	0.9	0.85	0.8	0.83
	PEOSPERous	0.82	0.33	0.23	1	0.38
	HIVcleave	0.73	0.61	0.69	0.67	0.68
	iProt-Sub	0.68	0.41	0.41	0.26	0.32
	DeepCleave	0.46	0.21	0.13	0.14	0.13
impensDataset	PU-HIV	**0.92**	**0.75**	0.73	0.65	**0.69**
	PU-HIV with standard SVM	**0.92**	0.74	0.71	0.62	0.67
	EvoCleave	0.88	0.64	0.77	0.42	0.54
	Rögnvaldsson et al. (2015)	0.9	0.7	0.69	0.62	0.65
	PROSPERous	0.83	0.17	0.16	1	0.27
	HIVcleave	0.56	0.29	0.29	0.45	0.35
	iProt-Sub	0.72	0.36	0.43	0.34	0.38
	DeepCleave	0.45	0.14	0.14	0.34	0.2
schillingDataset	PU-HIV	**0.94**	**0.75**	0.73	0.67	**0.7**
	PU-HIV with standard SVM	0.92	0.7	0.62	0.68	0.65
	EvoCleave	0.78	0.36	0.5	0.2	0.28
	Rögnvaldsson et al. (2015)	0.93	0.68	0.66	0.66	0.66
	PROSPERous	0.88	0.15	0.14	0.95	0.24
	HIVcleave	0.59	0.34	0.31	0.41	0.35
	iProt-Sub	0.75	0.37	0.39	0.34	0.37
	DeepCleave	0.52	0.13	0.13	0.43	0.2

*For each dataset, the best results are bolded.

Regarding the performance of PU-HIV in terms of AUC, we found that PU-HIV yielded the largest AUC scores in all the datasets except for 301Dataset where PU-HIV ranked as the second best algorithm and was only worse than HIVcleave. The average AUC score obtained by PU-HIV was better by 7.83, 2.38, 10.71, 36.7, 31.48, and 105.95% than EvoCleave, Rögnvaldsson et al. (2015), PROSPERous, HIVcleave, iProt-Sub, and DeepCleave, respectively. Another point worth to noting was the performance of HIVcleave in terms of AUC, as the performance of HIVcleave in the other datasets was not as good as in 301Dataset. In particular, HIVcleave performed the worst in impensDataset and schillingDataset, and it was the second worst algorithm in 746Dataset and 1625Dataset. To explain the reason for the contrasting performance of HIVcleave between 301Dataset and the other datasets, we conducted an in-depth investigation to the prediction results of HIVcleave and two things were observed. First, HIVcleave only provided the prediction results for 69 octapeptides in 301Dataset and accordingly the rejection rate of HIVcleave was as large as 77%. Second, for cleavable octapeptides that were correctly predicted by HIVcleave, the probabilities for most of them were below 0.5, thus yielding a smaller score of Recall as indicated in Table 3. Hence, HIVcleave was only able to make a prediction for octapeptides it could handle and moreover the confidence of HIVcleave on its prediction results was not as strong as that of PU-HIV.

We also noted that the Recall performance of PU-HIV was not as good as its Precision performance. Since the biased SVM classifier adopted by PU-HIV considers the unknown octapeptides as unlabeled samples instead of negative ones, it is difficult for PU-HIV to misclassify known cleavable octapeptides as negative due to the higher risk posed by the tolerance on the training errors generated by unlabeled octapeptides. In this regard, PU-HIV is very careful about the prediction of cleavable octapeptides and thus less known cleavable octapeptides are identified by PU-HIV. As a result, the Recall performance of PU-HIV performed worse than the Precision performance of PU-HIV.

The performance of PU-HIV in terms of PR-AUC and F-measure could be a strong indicator to demonstrate the promising accuracy of PU-HIV in predicting HIV-1 PR cleavage sites, as PU-HIV yielded the largest PR-AUC and F-measure scores for each dataset. Moreover, when compared with the AUC scores, a bigger margin was generated by PU-HIV for the PR-AUC and F-measure scores. A possible reason for that phenomenon was ascribed to the imbalance between positive and unlabeled octapeptides in most of datasets. Recall that the intuition of adopting the PR ROC analysis was to objectively measure the performance of each algorithm in imbalanced datasets. The superior performance of PU-HIV in terms of PR-AUC further verified that PU-HIV was more robust to the imbalance situation encountered in the training.

Moreover, comparing PU-HIV with iProt-Sub that integrated heterogeneous sequence and structural features, we noted that PU-HIV was never worse than any of them. Hence, the consideration of PU learning theory could allow prediction models solely resting on substrate sequences outperform those integrating different sources of biological information. On the other side, a possible reason for the poor performance of iProt-Sub was that combining different sources of biological information may, to some extent, confuse the prediction model, thus negatively affecting the accuracy performance. In this regard, an effective combination of features extracted from a single biological source, such as the sequence information considered in this work, was more useful for improving the performance of predicting HIV-1 PR cleavage sites.

To better indicate the improvement resulted from the adoption of biased SVM, we additionally developed a variant of PU-HIV with a standard SVM to perform the prediction task. According to Table 3, we noted that the average scores of AUC, PR-AUC, and F-measure obtained by PU-HIV were better by 0.86, 2.57, and 3.27%, respectively than those obtained by the standard version of SVM. This could also be a strong indicator that the performance of HIV-1 cleavage site prediction can be improved by considering the unknown octapeptides as unlabeled samples instead of negative ones.

Overall, across all datasets, the experiment results demonstrated the promising accuracy of PU-HIV in predicting HIV-1 PR cleavage sites, as it yielded the best average performance in terms of AUC, PR-AUC, and F-measure.

### 3.3. Cross Data Validation

To investigate the prediction accuracy of PU-HIV across different datasets, we conducted the experiments by alternatively training PU-HIV from one dataset and testing it on the other four datasets. The experiment results are given in Table 4.

**Table 4:** Experiment results of crossdata.

**Training set**	**Testing set**	**AUC**	**PR-AUC**	**F-measure**
301Dataset	746Dataset	0.94	0.94	0.87
	1625Dataset	0.93	0.78	0.76
	impensDataset	0.81	0.55	0.54
	schillingDataset	0.84	0.44	0.49
746Dataset	301Dataset	0.99	0.98	0.98
	1625Dataset	0.99	0.97	0.9
	impensDataset	0.84	0.63	0.6
	schillingDataset	0.89	0.56	0.56
1625Dataset	301Dataset	0.99	0.98	0.97
	746Dataset	0.98	0.98	0.96
	impensDataset	0.82	0.59	0.5
	schillingDataset	0.88	0.54	0.44
impensDataset	301Dataset	0.94	0.8	0.7
	746Dataset	0.89	0.9	0.75
	1625Dataset	0.89	0.71	0.63
	schillingDataset	0.94	0.71	0.66
schillingDataset	301Dataset	0.96	0.88	0.77
	746Dataset	0.93	0.94	0.87
	1625Dataset	0.94	0.8	0.72
	impensDataset	0.9	0.75	0.64

It was seen from Table 4 that the performance of PU-HIV taking 301Dataset, 746Dataset, and 1625Dataset as the training set was better than that using the other two datasets for training. To investigate the reason of that phenomenon, we compared the octapeptides in these datasets and found that there was a large overlap among the datasets of 301Dataset, 746Dataset, and 1625Dataset. In particular, 299 octapeptides in 301Dataset was found in 746Dataset while 659 octapeptides in 746Dataset were covered by 1625Dataset. That is to say, the PU-HIV classifier trained by using one of 301Dataset, 746Dataset, and 1625Dataset could perfectly separate cleavable and non-cleavable octapeptides in the other two dataets, but it may possibly misclassify new octapeptides. It was also for this reason that the prediction performance of PU-HIV for impensDataset and schillingDataset was worse, as these two datasets shared few octapeptides with the other three datasets.

Besides, the performance of PU-HIV presented in Table 4 also indicated that it was of great significance to choose appropriate datasets for training and testing. The PU-HIV classifier could be overfitting if the training set shared many octapeptides with the testing set, and accordingly the prediction accuracy was not evaluated objectively. For all the five datasets used in the experiments, 301Dataset, 746Dataset, and 1625Dataset were generated by replacing individual amino acids in cleaved octapeptides with other amino acids while impensDataset and schillingDataset were derived from human proteins. Moreover, we noted that PU-HIV performed the worst on impensDataset and schillingDataset by using 1625Dataset and 301Dataset for training. In this regard, we should avoid using 1625Dataset and 301Dataset as the training set when studying the substrate specificity in human proteins. Lastly, if we would like to combine datasets to compose a larger one where more relevant sequence-based features can be learned by PU-HIV, it is suggested to merge 746Dataset, impensDataset, and schillingDataset. There were two reasons for this suggestion, one was that the performance of PU-HIV trained by using these three datasets was generally better than using the other datasets, and the other was the considerably less overlap among these three datasets.

### 3.4. Analysis of Feature Significance and Contribution

As mentioned before, three different feature sets (amino acid identities, coevolutionary patterns, and chemical properties) were used by PU-HIV to predict HIV-1 cleavage sites. To investigate the respective contributions made by these features as well as which features were more significant than others, we performed an in-depth analysis to these features, and then evaluated the significance of these features for improving the performance of PU-HIV.

For the sake of simplicity, the feature sets of amino acid identities, coevolutionary patterns and chemical properties were denoted as AAI, CoP, and CheP, respectively. Then we constructed feature vectors for octapeptides under different combinations of AAI, CoP, and CheP and recorded the average AUC, PR-AUC, and F-measure scores obtained by PU-HIV in the 10-fold CV. The results are given in Table 5. Regarding the performance of PU-HIV for individual feature sets, we noted that the variant of PU-HIV that only considered AAI yielded the best performance in terms of all metrics. That is to say, the orthogonal encoding of amino acid identities was more effective in capturing the characteristics of substrate specificity and this observation was also consistent with the conclusion made by Rögnvaldsson et al. (2015). When more than one feature sets were combined, the performance of PU-HIV was further improved. Among all the combinations of two feature sets, AAI + CheP outperformed the other two combinations. Even though PU-HIV yielded the best performance when combining all the three feature sets, we noted that the difference in the performance of PU-HIV between AAI + CheP and AAI + CheP + CoP was rather small, as AAI + CheP + CoP only achieved 1.06, 1.18, and 1.26% relative gains in AUC, PR-AUC, and F-measure, respectively compared to AAI + CheP. The prediction accuracy of CoP was not as remarkable as claimed in Hu et al. (2020a). A possible reason for such a considerable difference could be ascribed to the fact that only coevolutionary patterns extracted from cleavable octapeptides were used by PU-HIV to construct feature vectors of octapeptides.

**Table 5:** Experiment results of feature analysis.

**Feature**	**AUC**	**PR-AUC**	**F-measure**
AAI	0.94	0.82	0.77
CheP	0.91	0.76	0.7
CoP	0.82	0.63	0.54
AAI + CheP	0.94	0.85	0.79
AAI + CoP	0.94	0.83	0.78
CheP + CoP	0.91	0.79	0.71
AAI + CheP + CoP	0.95	0.86	0.8

*Three different types of features are used in different combinations to construct feature vectors, and then cross-validation is performed on five independent data sets. The experimental result is the average of five independent data sets*.

Another point worth noting was that the difference in AUC between AAI and AAI + CheP + CoP was not as significant as the difference in either PR-AUC or F-measure. In particular, both PR-AUC and F-measure are concentrated on measuring the performance from the perspective of known cleavable octapeptides. Regarding the features of CoP, we only applied EvoCleave to extract the coevolutionary patterns from known cleavable octapeptides. This strategy made PU-HIV more accurate in predicting known cleavable octapeptides, thus yielding a better performance in both PR-AUC and F-measure when compared with AUC.

Overall, for these three feature sets, the consideration of AAI was the more significant factor to improve the performance of PU-HIV while CoP contributed the least to separate cleavable and uncleavable octapeptides.

## 4. Discussion

To predict HIV-1 PR cleavage sites, traditional machine learning models typically train a binary classifier using cleavable octapeptides as positive set and unknown octapeptides as negative set. It is possible for the negative set that some cleavable, yet unknown, octapeptides may be contained in the negative set. Hence, the practical performance of trained classifiers does not perform as well as they could have due to the noisy in negative set. In this work, we have proposed a novel PU learning algorithm PU-HIV by considering unknown octapeptides as unlabeled set instead of negative one. PU-HIV first uses a comprehensive combination of three different feature sets extracted from substrate sequences to represent octapeptides into feature vectors. After that, PU-HIV adopts a biased formulation of LSVM to distinguish the penalties of incorrectly classifying octapeptides in the positive and unlabeled sets. Experiment results demonstrated that PU-HIV could better model the classification problem for HIV-1 PR cleavage site prediction as it achieved significantly better results than the state-of-the-art prediction models. Thus, the novel HIV-1 cleavage sites predicted by PU-HIV may contribute to develop HIV-1 PR inhibitors for the purpose of AIDS treatment.

There are several reasons for the superior performance of PU-HIV. First, our motivation is based on the fact that there may exist some cleavable octapeptides that have not been verified by laboratory experiments but categorized in the negative set. This fact is widely observed in many problems of bioinformatics, such as disease gene identification (Yang et al., 2012), kinase substrate prediction (Yang et al., 2016), and protein-protein interaction prediction (Hu and Chan, 2015, 2017) and the successful applications of PU learning in solving these problems have verified the advantage of PU learning when handling the unlabeled data. It is also for this reason that we decided to adopt the PU learning theory for predicting HIV-1 PR cleavage sites more accurately. As such, a biased formulation of LSVM is implemented to maximize the number of unlabeled octapeptides classified as negative. On the other hand, the error of misclassifying cleavable octapeptides in the positive set could be further constrained by assigning a larger penalty, i.e., *C*_1_. Secondly, an integration of feature sets from a single source was verified to be more useful than that from multiple sources. PU-HIV only integrates three different feature sets extracted from the sequences of octapeptides. According to the results presented in Table 4, these three feature sets were complementary to each other, thus overcoming the shift-variance problem existed in cleaved octapeptides. Furthermore, it was the consideration of all of them that yielded the best performance of PU-HIV. However, the iProt-Sub model was restrictive with predicting HIV-1 PR cleavage sites, as the integration of features from multiple resources could degrade the accuracy by confusing the prediction model. Thirdly, the selection of training data was also essential to determine the performance of PU-HIV. Among all prediction models used for comparison, we noted that the generalization ability of PU-HIV outperformed the others. That is to say, the negative influence induced by the noisy in unlabeled octapeptides could be minimized by the biased LSVM of PU-HIV. Lastly, according to Table 3, we realized that only using the ROC analysis may not be able to precisely indicate the accuracy of prediction models especially for those trained by imbalanced dataset. A large AUC score only revealed the ability of clearly separating cleavable and non-cleavable octapeptides, but it failed to measure the confidence of such separation. Taking the prediction model proposed by Rögnvaldsson et al. (2015) as an example, its performance in terms of AUC was close to that of PU-HIV, but its PR-AUC and F-measure scores were much less than those obtained by PU-HIV. After investigating the prediction results of Rögnvaldsson et al. (2015), we noted that the probabilities of many cleaved octapeptides were <0.5 and it was for this reason that the prediction model proposed by Rögnvaldsson et al. (2015) obtained smaller PR-AUC and F-measure scores. Hence, in addition to AUC, there is a necessity to consider the PR-AUC and F-measure for a more precise evaluation about the ability of predicting HIV-1 PR cleavage sites.

The computational cost of PU-HIV is composed of two parts, one is the time taken by feature vector construction and the other is the computational cost of biased SVM. Given that *n* is the size of the training set, the computational cost of feature vector construction is *O*(*n*), as we have to construct feature vectors for all samples in the training set. Regarding the biased SVM adopted by PU-HIV, its minimum computational cost for training is *O*(*n*^2^), which is identical to LSVM. Hence, the overall computational cost of PU-HIV is *O*(*n*^2^).

Regarding our future work, we would to incorporate the ensemble learning framework into PU-HIV. By employing multiple biased LSVM classifiers and combining their prediction results in an efficient manner, we expect to reduce the variance and improve the robustness of PU-HIV. Considering the different performance of PU-HIV on these datasets, there may be significant effects from using different feature encoding methods when we train PU-HIV on a particular dataset. Hence, we may explore more complicated machine learning methods for feature extraction and reduction to better describe the characteristics of cleavable and unlabeled octapeptides.

## Data Availability Statement

Publicly available datasets were analyzed in this study. This data can be found at: https://github.com/allenv5/PU-HIV.

## Author Contributions

ZL implemented the algorithms, carried out the experiments, and drafted the manuscript. LH conceived of the study, and participated in its design and coordination and helped to draft the manuscript. ZT and CZ performed the statistical analysis. All authors read and approved the final manuscript.

## Conflict of Interest

The authors declare that the research was conducted in the absence of any commercial or financial relationships that could be construed as a potential conflict of interest.
